# Isobutyramide and Slow-Release Urea as Substitutes for Soybean Meal in the Finishing Diet of Beef Cattle

**DOI:** 10.3390/ani14091321

**Published:** 2024-04-28

**Authors:** Chen Wei, Haiying Tao, Guifen Liu, Kechuan Tian

**Affiliations:** 1 Institute of Animal Science and Veterinary Medicine, Shandong Academy of Agricultural Sciences, Jinan 250100, China; weichenchen1989@126.com (C.W.); yinghaitao@163.com (H.T.); 2 Shandong Key Lab of Animal Disease Control and Breeding, Jinan 250100, China

**Keywords:** isobutyramide, slow-release urea, soybean meal, beef cattle

## Abstract

**Simple Summary:**

Soybean meal is one of the main high-quality protein feedstuffs and deficient in China, and the sustainability of the beef cattle industry is facing more and more challenges. It is essential to seek efficient alternative N sources to ensure a steady increase in beef production. The in vitro and in vivo experiments were conducted to test if dietary isobutyramide and slow-release urea could benefit animal performance, rumen fermentation, nutrient digestibility, and blood parameters and develop a new strategy for substituting soybean meal in beef cattle. We suggest the optimal strategy is the isonitrogenous substitution of soybean meal with 0.3% slow-release urea and 0.6% isobutyramide of the diet.

**Abstract:**

Two experiments were conducted to investigate the effects of isobutyramide (IBA) and slow-release urea (SRU) as substitutes for soybean meal (SBM) in the finishing diet of beef cattle. The completely randomized design in vitro experiment with five treatments, i.e., control, 0.9% SRU group, 0.6% SRU + 0.3% IBA group (SRU-I), 0.3% SRU + 0.6% IBA group (IBA-S), 0.9% IBA group was conducted. The results showed that the IBA-S and IBA increased (*p* ≤ 0.05) substrate disappearance of dry matter (DM), neutral detergent fiber (NDF), acid detergent fiber (ADF), total gas, and total volatile fatty acids (TVFA). The SRU group had the highest (*p* < 0.01) crude protein disappearance and ammonia nitrogen concentration, but the IBA contrarily decreased (*p* < 0.01) them compared with the control. Inclusion of IBA increased isobutyrate concentrations (*p* = 0.01) with the highest value for the IBA group. Then, an 84-day replicate 4 × 4 Latin square design with 8 Angus steers and four treatments, i.e., control, SRU, SRU-I, IBA-S was performed. The results showed that the treatments did not affect DM intake (*p* > 0.05) but tended (*p* = 0.09) to increase average daily gain. The inclusion of IBA increased (*p* < 0.05) the apparent digestibility of DM, organic matter, NDF, ADF, TVFA, and microbial crude protein with the highest values for the IBA-S group. The IBA-contained groups also increased (*p* ≤ 0.01) isobutyrate concentration, activities of carboxymethyl cellulase and xylanase, and the relative abundance of *Butyrivibrio fibrisolvens* with the highest values for the IBA-S group. The SRU had no effect on animal growth and nutrient apparent digestibility. In conclusion, IBA was developed as a new substitute for SBM in the finishing diet of beef cattle, and the optimal strategy was the isonitrogenous substitution of SBM with 0.3% SRU and 0.6% IBA of the diet.

## 1. Introduction

With the increasing demand and limited production of soybeans, China has become the world’s top consumer and largest importer, of which the volume of import has reached approximately 100 million tons/year in recent years [1]. The imported soybeans are mainly crushed to obtain soybean oil and soybean meal (SBM) used as animal feed. With the increase in population and economic development in China, the demand for high-quality protein, such as beef, is increasing rapidly, which has led to a large increase in the number of beef cattle raised. Soybean meal is widely used as a common source of crude protein (CP) in the growing and finishing phases of beef cattle in China. However, with its increasing price and insufficiency, the sustainability of the beef cattle industry is facing more and more challenges [2,3]. Thus, it is essential to seek efficient alternative N sources to substitute SBM and ensure a steady increase in beef production.

Ruminants have the unique ability to efficiently utilize non-protein nitrogen (NPN), which can be transformed by rumen microbes to microbial crude protein (MCP) and ultimately used by the host animals [4]. The most common NPN source used in ruminant production is urea, due to its commercial availability with low cost and high N content. Feeding urea directly can cause its rapid ruminal decomposition, which leads to inefficient utilization and hyperammonemia [5]. Slow-release techniques such as gelatinized starch and fatty acid coating are applied to solve the problem. Although slow-release urea (SRU) has been widely studied and used as NPN additives in ruminant diets, the effects of SRU replacing SBM on feed intake, nutrient digestibility, or animal performance in beef cattle remain controversial due to different doses, slow-release methods, or feeding phases [6,7,8]. The other NPN source is water-soluble amide-N compounds such as nicotinamide, glutamine, and fatty acyl amide, which can be used by rumen microbes to synthesize MCP [9]. Isobutyramide (IBA) is a typical fatty acyl amide that chemically links isobutyryl through an amide bond to an amino group. Pure IBA has less N content (16.1%) than that of urea (46.7%), but IBA may be decomposed in rumen to isobutyrate which has been reported to improve rumen fermentation and nutrient digestibility by increasing microbial proliferation and enzyme activity [10,11]. Hence, IBA may be a potential NPN source that both provides utilizable N and improves feed efficiency.

Limited studies were focused on using IBA and SRU (separated or combined) as potential substitutes for SBM in the finishing diet of beef cattle. Therefore, the in vitro and in vivo experiments were conducted to test if dietary IBA and SRU could benefit animal performance, rumen fermentation, nutrient digestibility, and blood parameters and develop a new strategy for substituting SBM in beef cattle.

## 2. Materials and Methods

### 2.1. In Vitro Experiment

#### 2.1.1. Experimental Design, Treatments and Donor Animals

The experiment was repeated 3 times as a completely randomized design with 5 treatments and 6 replicates for each treatment. Isobutyramide (C_4_H_9_NO, purity > 98%, Hubei Xin Runde Chemical Industry Co., Ltd., Wuhan, China) and SRU (CH_4_N_2_O, purity = 73% (corn and bentonite, 26%; NaCl, 1%), Henan Hongyuan Feed Co., Ltd., Zhengzhou, China) were used as alternative N sources to substitute SBM. The release rate of SRU was 69.7% at 2 h and 91.9% at 6 h of the in vitro incubation. According to a meta-analysis study of SRU supplementation trials, the average SRU dosage was 0.88% (dry matter, DM) of diet [12]. Hence, the isonitrogenous substituting ratio was set as 0.9%, and the treatment groups were control, 0.9% SRU group (SRU), 0.6% SRU + 0.3% IBA group (SRU-I), 0.3% SRU + 0.6% IBA group (IBA-S), 0.9% IBA group (IBA). Three rumen-fistulated beef steers (Limousine × Luxi yellow cattle, bodyweight: 465 ± 22 kg) fed whole corn silage (DM: 28.6%; Organic matter (OM): 95.0%; Ether extract (EE): 3.4%; CP: 7.8%; Neutral detergent fiber (NDF): 40.0%; Acid detergent fiber (ADF): 22.6%) ad libitum was employed as donor animals for rumen contents. The contents were sampled from different sites of the rumen at 7:30 a.m. before morning feeding. After collection, the contents were filtered through 4 layers of gauze. An equal volume of rumen fluid from each animal was mixed well in a prewarmed air-tight thermos.

#### 2.1.2. Incubation and Sampling

Before incubation, 0.7 g ground substrate (Table 1, DM basis) of each treatment was added into each labeled and pre-weighed Ringbio filter bag (F25, Ringbio instrument group Co., Ltd., Salford, Greater Manchester, UK), which was placed into serum bottle after heat-sealed. A sufficient anaerobic medium was prepared the morning before incubation in accordance with Goering and Van Soest [13]. Then, 45 mL prewarmed medium and 15 mL inoculum (3:1 ratio) were dispensed anaerobically to the 125 mL bottles by continuously flushing with O_2_-free CO_2_. After sealing with a 14 mm butyl rubber stopper plus an aluminum crimp cap, the bottles were incubated on a shaker with 125 oscillations/min at 39 °C for 24 h.

In the process of incubation, headspace gas pressure from each bottle was measured by a traceable manometer (model 06-664-21, Fisher Scientific Inc., Pittsburgh, PA, USA) after 3, 6, 9, 12, 18, and 24 h of incubation. After 24 h of incubation and gas sample collection, the serum bottles were immediately placed on ice to terminate fermentation. Filter bags from each bottle were removed and rinsed thoroughly with cold water until the effluent ran clear and dried at 55 °C for 48 h to determine DM disappearance (DMD). Feed residues from 3 filter bags per treatment were analyzed for NDF and ADF to calculate NDF (NDFD) and ADF disappearance (ADFD). For the other 3 filter bags, feed residues were used for CP analysis to calculate CP disappearance (CPD). After the pH values of the fermentation fluids were measured by a pH meter (HI 9125, Hanna Instruments Inc., Smithfield, RI, USA), 1 mL supernatant of each bottle was mixed with 0.2 mL of 25% (*w*/*v*) metaphosphoric acid and kept at −20 °C for volatile fatty acid (VFA) test. Another 1 mL supernatant was combined with 0.2 mL of 1% (*w*/*v*) H_2_SO_4_ and kept at −20 °C for the ammonia nitrogen (NH_3_-N) test.

#### 2.1.3. Chemical Analysis

The substrate was determined for the DM, ash, and EE following methods 934.01, 924.05, and 920.39 of AOAC [14]. The total N of substrate and feed residues were determined by the method of Kjeldahl and CP was equal to the N value multiplied by 6.25. The NDF and ADF contents were determined using a Ringbio Fibre Analyser (R-200, Ringbio instrument group Co., Ltd., Salford, Greater Manchester, UK) with reagents as described by Van Soest et al. [15], and values are expressed inclusive of ash. Heat stable α-amylase (A800732, Macklin Inc., Shanghai, China) and sodium sulfite was used during NDF analysis. The concentrations of VFA were measured using a gas chromatograph (Model 7890A, Agilent Technologies, Santa Clara, CA, USA) with a capillary column (30 m × 0.25 mm i.d. × 0.25 µm phase thickness, HP-INNOWax, Agilent Technologies, Santa Clara, CA, USA) and a flame-ionization detector. The oven temperature was initially kept at 60 °C for 1 min, increased by 10 °C/min to 200 °C, and held at 200 °C for 2 min. The temperatures of the injector and detector were 200 °C and 230 °C. The concentration of NH_3_-N was determined using a spectrophotometer (Ultrospec-3100, Amersham Biosciences, Piscataway, NJ, USA) according to the method of Chaney and Marbach [16].

### 2.2. In Vivo Experiment

#### 2.2.1. Animal, Management and Experimental Design

Eight healthy Angus steers aged 14 months, with an average initial body weight of 477 ± 14 kg, were used in the experiment. The cattle were housed in individual pens and well cared for. The diets were identical to the substrates of the in vitro experiment and were provided twice daily ad libitum at 7:30 a.m. and 17:30 p.m., respectively. The diets met the nutrient requirements of beef cattle, according to Feng [17], and fresh drinking water was available all the time. According to the results of the in vitro experiment, the in vivo experiment was performed as a replicate 4 × 4 Latin square design with the treatment groups including control, SRU, SRU-I, and IBA-S. The treatments were prepared when formulating the concentrates, which were then mixed well with roughages every meal before being fed to the cattle. The experiment lasted 84 days with 4 periods, and every period had 21 days, including 17 days for adaptation and subsequent 4 days for sampling.

#### 2.2.2. Sampling

At the beginning of the experiment and the end of each period, the cattle were individually weighed and recorded before morning feeding.

During 18–21 days of each period, the feces of each animal were respectively and totally collected using plastic buckets and shovels after they defecated. The collection process was inspected by special personnel day and night. At 10:00 a.m. of every sampling day, about 1% of fecal samples of each cattle were individually taken after being measured and blended. The other 200 g fecal samples were sampled with 40 mL 10% (*v*/*v*) tartaric acid for N analysis. Diets were offered, and orts were weighed daily for each cattle. The feedstuff samples were taken every day and pooled together within each period. All the samples were kept at −20 °C for later analysis.

On days 18 and 19 of each sampling period, the rumen fluids of each cattle were obtained through the esophagus using a tube sampler at 9:30 a.m. The first sample of rumen fluid was discarded to avoid saliva contamination. Then, about 400 mL of rumen fluid was yielded and mixed well, and the pH was measured immediately by a pH meter (HI 9125, Hanna Instruments Inc., Smithfield, RI, USA). Fifty mL subsamples of each cattle were sampled and kept at −20 °C for later analysis. Twenty mL subsamples were kept in liquid N for ruminal microbe analysis. Ten mL subsamples were preserved with 10 mL methyl green-formalin-saline solution and stored in darkness at room temperature until protozoa enumeration. Another 10 mL subsamples were separated and handled as described by Hristov et al. [18] for analyzing the activities of carboxymethyl cellulase and xylanase.

On days 20 and 21 of each sampling period, 20 mL blood samples were collected from each cattle at 9:30 o’clock using vacutainers containing K_2_-EDTA (Aosaite Medical Instrument Co., Ltd., Heze, China) through the jugular vein and were then centrifuged at 2000 rpm for 15 min at room temperature to obtain plasma. The samples were kept at −20 °C for later analysis.

#### 2.2.3. Chemical Analysis

After drying at 65 °C for 48 h, the feedstuff samples were pulverized by a cutting mill, and the fecal samples were roughly ground using mortar and pestle and pooled together per cattle per period. All the ground samples were sieved through a 2 mm mesh for subsequent analysis. The methods for analyzing DM, ash, CP, NDF, and ADF of the feedstuffs and feces were the same as the in vitro experiment.

Rumen fluids thawed at room temperature and homogenized. The subsamples for VFA and NH_3_-N determination were prepared and measured in the same way as those of the in vitro experiment. Ten mL subsamples were centrifuged at 4000 rpm for 15 min to obtain supernatant as the isolated microbial samples. Then, the MCP was measured by trichloroacetic acid precipitation method described by Wang et al. [19]. Protozoa were counted by optical microscopy (BK300, Optec instrument Co., Ltd., Chongqing, China) and a counting chamber (Qiujing biochemical reagent & instrument Co., Ltd., Shanghai, China). The activities of rumen carboxymethyl cellulase and xylanase were measured by the procedure of Patra et al. [20], and the released reducing sugars (glucose/xylose) were determined by colorimetric kits (F006/A035, Nanjing Jiancheng Bioengineering Institute, Nanjing, China).

The total bacterial 16S rDNA was extracted from a homogenized rumen fluid sample using the QIAamp DNA Stool Mini Kit (51604, Qiagen, Hilden, Germany) according to the corresponding handbook with a few modifications, i.e., the treatment after adding InhixitEX Buffer: 95 °C for 10 min; the volume of adding ATE solution: 100 μL; ATE solution diluting time: 5 min. The concentration and purity were measured using a NanoDrop spectrophotometer (2000, Thermo Scientific, Wilmington, DE, USA) at the optical density of 260 and 280 nm. The primers of the total bacteria [21], fungi [21], *Ruminococcus albus* (*R. albus*) [22], *Ruminococcus flavefaciens* (*R. flavefaciens*) [21], *Fibrobacter succinogenes* (*F. succinogenes*) [21], Butyrivibrio fibrisolvens (*B. fibrisolvens*) [23] and *Prevotella* [23] were listed in Table 2. The real-time quantitative polymerase chain reaction (qPCR) was performed using a LightCycler platform (480 II, Roche Inc., Basel, Switzerland) with fluorescence detection of SYBR green dye (RR420A, Takara Bio Inc., Dalian, China). The volume of the qPCR mixture was 20 μL consisting of 10 μL SYBR Premix Ex Taq, 0.4 μL forward primer (10 μmol/L), 0.4 μL reverse primer (10 μmol/L), 2 μL DNA template, and 7.2 μL sterile distilled water. The measuring procedure was initiated at 95 °C for 30 s, followed by 40 cycles at 95 °C for 5 s and 60 °C for 30 s, finished by generating a melting curve at 95 °C for 5 s, 60 °C for 1 min and 95 °C for 15 s.

The plasma parameters were measured by an automatic biochemical analyzer (7100, Hitachi Ltd., Tokyo, Japan). Blood urea nitrogen (BUN), total protein (TP), glucose (GLU), triglyceride (TG), cholesterol (CHO), alanine aminotransferase (ALT), aspartate Aminotransferase (AST), creatinine (CREA) and uric acid (UA) in plasma were determined by urease-GLDH method, biuret method, hexokinase method, GPO-PAP method, CHOD-PAP method, alanine substrate method, aspartic acid substrate method, sarcosine oxidase method and uricase method using colorimetric kits (MedicalSystem Biotechnology Co., Ltd., Ningbo, China), respectively. Trimethylamine oxide (TMAO) in plasma was precipitated by Reinecke’s salt under strongly acidic conditions (pH = 1, 12 M of HCl). Then, the precipitate was dissolved in 70% acetone (*v*/*v*) and determined at 525 nm by a spectrophotometer (UV-3200, Mapada Instrument Co., Ltd., Shanghai, China).

### 2.3. Calculation and Statistical Analysis

Pressure values, corrected for the gas produced from negative controls, were employed to estimate gas volume using the equation of Mauricio et al. [24]:

Gas volume (mL) = 0.18 + [3.697 × gas pressure (psi)] + [0.0824 × gas pressure^2^ (psi)]

The gas production kinetics were calculated using the model of France et al. [25]:

y = b × [1 − e^−c(t − L)^]

where y = the volume of gas at time t (h); b = the asymptote gas production (Vmax; mL/g DM); c = fractional degradation rate (S;/h); and L = lag phase (L; h).

The PCR efficiency (E) was determined from the slope of the external calibration curve in line with the equation: E = 10 ^(−1/slope)^ − 1. The abundance of the microorganisms was exhibited as a proportion of total rumen bacterial 16S rDNA using the equation:

Relative abundance of target = 2^−(Ct target − Ct total bacteria)^

where Ct means the threshold cycle.

The data were subjected to analysis of variance using the MIXED procedure of SAS 9.1 (SAS Institute Inc., Cary, NC, USA). For the in vitro experiment, the mixed model included the fixed effect of treatment and the random effect of repeated time. For the in vivo experiment, the protozoa population was transformed to log_10_ values before analysis to assume normality. The mixed model included the fixed effect of treatment and the random effect of the animal and experimental period. The Kenward-Roger option was used to adjust degrees of freedom. Results were displayed as least-square means, and the PDIFF option was used to compare differences among treatment groups. Difference was declared as statistically significant at *p* ≤ 0.05 and tendency was discussed at 0.05 < *p* ≤ 0.10.

## 3. Results

### 3.1. In Vitro Experiment

After 24 h incubations, the SRU had no effect (*p* > 0.05) on DMD, NDFD, ADFD, and total gas compared with the control (Table 3) but had the highest CPD followed by SRU-I, and IBA contrarily decreased CPD (*p* < 0.01). Substituting SBM by the SRU-I, IBA-S, and IBA significantly increased (*p* < 0.01) DMD, NDFD, total gas, and Vmax (*p* = 0.02) compared with the control and SRU, and the IBA-S and IBA had the highest values. The IBA-S and IBA also increased ADFD (*p* = 0.05) compared with the control, SRU and SRU-I. The treatments had no effect on gas production kinetics index S (*p* = 0.74) and L (*p* = 0.28).

For the fermentation parameters, the IBA-S and IBA had lower (*p* < 0.01) pH than that of the control and SRU, and no difference was observed between SRU-I and other groups. The treatments exerted a similar effect (*p* < 0.01) on NH_3_-N concentration compared with CPD. The treatments affected total volatile fatty acids (TVFA) (*p* < 0.01), acetate concentration (*p* < 0.01), and acetate to propionate (A:P) ratio (*p* = 0.01) in the same way as that of DMD. Concentrations of propionate (*p* = 0.34), valerate (*p* = 0.97) and isovalerate (*p* = 0.67) were not affected by the treatments. Compared with the control, the IBA inclusion groups tended (*p* = 0.09) to increase butyrate concentration. Compared with the control and SRU, the IBA had the highest isobutyrate concentration, followed by the IBA-S and SRU-I (*p* = 0.01).

### 3.2. In Vivo Experiment

Dry matter intake (DMI) was not affected by the treatments (*p* = 0.71) (Table 4). Compared with the control and SRU, the SRU-I and IBA-S tended (*p* = 0.09) to increase the average daily gain (ADG). The apparent digestibility of DM (*p* = 0.03), OM (*p* = 0.01), NDF, and ADF (*p* < 0.01) was respectively enhanced by the SRU-I and IBA-S compared with the control and SRU, with the highest values for the IBA-S. The apparent digestibility of CP did not differ among the groups (*p* = 0.49).

Substituting SBM by the SRU-I and IBA-S increased (*p* = 0.02) ruminal pH compared with the control and SRU (Table 5). The SRU had the highest NH_3_-N concentration, followed by SRU-I compared with the control and IBA-S (*p* < 0.01). The treatments changed concentrations of TVFA (*p* = 0.02), acetate (*p* = 0.04), butyrate (*p* = 0.03), isobutyrate (*p* = 0.01), A:P ratio (*p* = 0.01) and MCP (*p* < 0.01) in the same way to that of apparent digestibility of DM. Concentrations of propionate (*p* = 0.90), valerate (*p* = 0.52), isovalerate (*p* = 0.77) and protozoa numbers (*p* = 0.16) were not affected by the treatments. Compared with the control and SRU, the activities of carboxymethyl cellulase and xylanase were significantly enhanced (*p* < 0.01) by the SRU-I and IBA-S, with the highest values for the IBA-S.

The relative abundances of fungi (*p* = 0.73), *R. albus* (*p* = 0.76), *R. flavefaciens* (*p* = 0.99), *F. succinogenes* (*p* = 0.82), and *Prevotella* (*p* = 0.96) did not differ among the groups (Table 6). Compared with the control and SRU, the SRU-I and IBA-S significantly increased (*p* < 0.01) the relative abundances of *B. fibrisolvens*, with the highest value for the IBA-S.

For the blood parameters, the SRU and SRU-I increased BUN (*p* = 0.03) compared with the control and IBA-S (Table 7). GLU was increased (*p* = 0.03) by the SRU-I and IBA-S in comparison with the control and SRU. Other blood nutrient indexes such as TP (*p* = 1.00), TG (*p* = 1.00), and CHO (*p* = 0.99) did not differ among the groups (*p* > 0.05). The health indexes CREA (*p* = 0.99), UA (*p* = 1.00), TMAO (*p* = 0.75), ALT (*p* = 0.79), and AST (*p* = 0.87) were also not affected by the treatments.

## 4. Discussion

As SBM is one of the main high-quality protein feedstuffs and is relatively deficient in China, developing SBM substitutes is highly necessary, and the availability, price, efficiency, and safety of the substitutes need to be generally taken into account. In the present study, the substitute SRU was gelatinized starch–urea, and IBA was analogous to isobutyric acid, but the carboxyl group was replaced by the amino group. They were both commercially available. For the price, SRU cost ~2500 ¥ (393 $)/t, which was much lower than that of SBM (~3500 ¥ (484 $)/t). Although IBA was ~6000 ¥ (831 $)/t, isonitrogenous IBA (N content, 16.1%) cost less than SBM (N content, ~7.5%). Their safety and efficiency were tested by replacing SBM with single or combined substitutes in an isonitrogenous manner.

The effects and proper dosage of the substitutes on rumen fermentation were tested by the in vitro experiment. As a slow rumen-released N compound, SRU can potentially benefit rumen function by improving the synchrony of fermentable energy and N in the rumen [26], but 0.9% SRU substitution in the present study made no difference, which was consistent with Salami et al. [8] who observed replacing SBM with SRU up to 1.3% (as-fed) did not affect in vitro rumen fermentation. It seemed that the available energy might be a limited factor for the absent response as the SRU was hydrolyzed faster than SBM according to the CPD and NH_3_-N concentration. For the IBA group, the lowest CPD and NH_3_-N concentration indicated that IBA was also a slow rumen-released NPN, and the possible reason might be amide bond of fatty acyl amides is resistant to rumen microbial degradation [27]. Thus, the CPD and NH_3_-N concentrations were decreased with increasing content of IBA in the substrates. Compared with the control and SRU group, the IBA inclusion treatments improved rumen fermentation by increasing DMD and fiber disappearance. The greater total gas, Vmax, TVFA concentration, and lower pH value also approved the beneficial function of IBA. In addition, more acetate, butyrate productions, and altered rumen fermentation patterns from propionate to acetate were in line with more fiber degradation. Brand-chained VFAs such as isobutyrate and isovalerate are generally derived from the deamination of branched-chain amino acids in the rumen [28]. Compared with the control, the IBA group had less substrate branched-chain amino acids content and CPD; hence, the present results indicated that IBA could be ruminally degraded to isobutyrate, which was required for ruminal fiber-degrading microorganisms [29] and could increase bacterial cellulolytic activity [10,30]. Liu et al. [31] reported that supplementation of isobutyrate in the diet of steers increased in situ ruminal degradability of feed DM and NDF. Hence, isobutyrate derived from IBA might be the main reason to improve rumen fermentation. Compared with the IBA-S group, the separated addition of IBA as the substitute was excluded for further investigation as it exerted similar effects but had lower CPD and higher costs.

The in vivo experiment further clarified the effects of the substitutes on animal performance, digestion, and health. No difference in DMI among the groups indicated that no palatability problem of SRU or combined substitutes existed. Benedeti et al. [6] found that substituting SBM (9.2% or 11.2% of the diets) with SRU (urea coated with lipid sources) up to 100% (added 1.74% or 2.14% of the diets) linearly decreased DMI of finishing beef cattle. The divergence might be explained by the present substitute level (0.9%) being lower and different slow-release methods. A meta-analysis study reported that supplementing SRU at an average of 0.88% DM of the diet could increase live-weight gain in beef cattle [12], but isonitrogenous substitution of SBM by 0.9% SRU did not change animal growth in the present study. It suggested that SRU was likely to improve animal performance only by providing extra available N. As the DMI was similar, the response of ADG for the substitute groups was associated with the changes in nutrient digestibility. The increase in the apparent digestibility of DM, OM, NDF and ADF for the IBA inclusion groups was consistent with the in vitro results and indicated that the combined substitutes benefited digestion and production in the finishing phase of beef cattle. The positive effects could be due to the increased fibrolytic enzyme activities and relative abundance of *B. fibrisolvens* in the SRU-I and IBA-S groups. Isobutyrate was reported to be used as a precursor of branched-chain amino acids and fatty acids by ruminal microbes, especially cellulolytic bacteria [32,33]. Previous studies have found that isobutyrate could increase rumen and total tract digestions of DM and NDF in vitro or in vivo [10,30,31]. Hence, the present results indicated the increased isobutyrate that derived from IBA hydrolyzation stimulated the proliferation of *B. fibrisoluens* and enhanced the activities of carboxymethyl cellulase and xylanase for improving rumen fermentation and nutrient digestibility. The best beneficial effects for the IBA-S group could be due to the highest isobutyrate concentration. No response of the total tract apparent digestibility of CP agreed with no change of the proteolytic *Prevotella* proliferation, indicating that the total digestible N was similar among the groups, although the substitutes were decomposed faster than SBM in the rumen. Gardinal et al. [7] reported that partially substituting SBM with 1% SRU increased the total tract digestion of CP. The possible reason was that adding SRU in that trial increased CP content (control, 14.36% CP vs. SRU, 15.21% CP) rather than isonitrogenous substitution of SBM.

The in vivo rumen parameters indicated that substituting SBM with SRU alone did not affect rumen function, but the combined substitutes improved rumen fermentation, which was consistent with the in vitro results. The ruminal pH values among the groups ranged from 6.34 to 6.51, which were within 5.5–7.0, as expected for optimal rumen fermentation [34]. The decreased pH values for the SRU-I and IBA-S groups were in line with the corresponding TVFA productions that derived from the increased DM and fiber digestion. Compared with the control, the SRU and SRU-I substitutes increased the NH_3_-N concentration, which could be explained by the faster-hydrolyzed rate of SRU than SBM. Similar NH_3_-N concentration to the control suggested a higher level of dynamic balance between NH_3_-N production and utilization in the IBA-S group, as the latter MCP production was higher. As acetate and butyrate productions were highly related to structural carbohydrate degradation [35], the increased acetate, butyrate, and A:P ratio within the SRU-I and IBA-S groups could be due to the promoted fiber digestion caused by the IBA inclusion. In addition, the enhanced isobutyrate production for the combined substitute groups was in line with the in vitro experiment. Although the SRU resulted in the highest NH_3_-N concentration, no difference in MCP was found compared to the control, whereas the combined substitutes promoted MCP synthesis. The possible reason was that the inclusion of IBA facilitated rumen microbial synchronization between fermentable energy and available N by both increasing the apparent digestibility of OM and NH_3_-N concentration. Protozoa were able to predate on bacteria [36], but no response of protozoa numbers was observed with the increased MCP synthesis. It might be due to an offset under the beneficial and detrimental conditions, as the increased isobutyrate concentration could also inhibit ruminal protozoa proliferation [10]. Plant NDF is mainly composed of cellulose, hemicellulose and lignin, of which the former two parts are major plant cell wall polysaccharides that can be decomposed by rumen microbial enzymes. Carboxymethyl cellulase and xylanase are typical rumen fibrolytic enzymes to hydrolyze cellulose and xylan (the main component of hemicellulose) [37,38,39]. Fibrolytic carboxymethyl cellulase and xylanase could be produced by ruminal cellulolytic bacteria such as *B. fibrisoluens* [40]. The present results indicated that the IBA inclusion treatments stimulated the fibrolytic enzyme activities, which could be due to the beneficial effects of the produced isobutyrate on the growth of *B. fibrisolvens*. It should be noted that directly adding 16.8 g or 25.2 g isobuyrate/d/steer increased not only *B. fibrisolvens* proliferation but also other cellulolytic microbes such as fungi, *R. albus*, *R. flavefaciens*, *F. succinogenes* [10], which was inconsistent with the present results. As the ruminal isobutyrate concentration was not given by Wang et al. [10], the possible reason might be that *B. fibrisolvens* was more sensitive to the isobutyrate changes or the isobutyrate derived from IBA was insufficient to benefit the growth of other cellulolytic microbes.

Animal physiological change and health can be monitored and evaluated by blood biochemical profiles [41]. Compared with the control, the nutrient and health indices for the substitute groups were within the physiological range of the steers, indicating no detrimental effects existed. Generally, higher ruminal NH_3_-N concentration resulted in higher BUN concentration. The present study observed a similar change between the ruminal NH_3_-N and BUN among the groups. Salami et al. [8] reported that substituting SBM by up to 3% SRU did not affect the BUN concentration of the growing beef cattle. The divergence could be due to their slower released urea, resulting in no change of ruminal NH_3_-N concentration. Animal nutritional level and body protein status can be reflected by blood TP level, which was similar among the groups and consistent with the corresponding total tract CP digestion. It suggested that isonitrogenous substitution of SBM by the substitutes did not decrease body N supply in the finishing period of beef cattle. However, Salami et al. [8] showed that using 1% or 3% SRU as a substitute for SBM decreased serum TP of the growing beef cattle. It might be attributed to different requirements for the CP sources in beef cattle at different growth phases. Blood GLU is one of the indicators to reflect body energy level and generally has in positive relationship with ruminal propionate production. Substituting SBM with the combined substitutes enhanced the plasma GLU level but did not change the propionate concentration. The possible reason might be the contribution of glucogenic amino acids that are derived from the microbial protein [42]. The plasma TG and CHO concentrations reflect body lipid metabolism levels, and the results showed that the substitutes had nothing to do with lipid metabolism in beef cattle. The health indices, including the CREA, UA, TMAO, AST, and ALT, are common indicators to reflect if animal tissue metabolism and liver and kidney function were normal [43,44]. The present results suggested that replacing SBM with the present level of the substitutes did not induce health issues in beef cattle.

## 5. Conclusions

The present work developed IBA as a new substitute for SBM in beef cattle. Both IBA and SRU were efficient alternative N sources for partially substituting SBM in the finishing diet of beef cattle. As the combination of IBA and SRU showed beneficial effects on animal performance and nutrient digestibility without compromising feed intake and health of beef cattle, the optimal strategy recommended was the isonitrogenous substitution of SBM with 0.3% SRU and 0.6% IBA of the diet.

## Figures and Tables

**Table 1:** Chemical composition of total mixed diets (DM basis).

Items ^1^	Control	SRU	SRU-I	IBA-S	IBA
Ingredients, g/kg					
Corn	270	270	270	270	270
SBM	150	91	102.5	113.5	125
Wheat bran	50	100	88.5	77.5	66
SRU	0	9	6	3	0
IBA	0	0	3	6	9
NaCl	10	10	10	10	10
Sodium bicarbonate	10	10	10	10	10
Premix ^2^	10	10	10	10	10
Whole corn silage	300	300	300	300	300
Wheat straw	200	200	200	200	200
Nutrient levels					
DM (g/kg)	716	717	716	715	715
OM (g/kg DM)	937	938	938	936	936
EE (g/kg DM)	23.5	23.9	23.6	23.7	23.9
CP (g/kg DM)	131	131	131	131	131
NDF (g/kg DM)	365	379	376	372	370
ADF (g/kg DM)	204	211	210	209	207

^1^ SRU, 0.9% slow-release urea group; IBA, 0.9% isobutyramide group; SRU-I, 0.6% slow-release urea + 0.3% isobutyramide group; IBA-S, 0.6% isobutyramide + 0.3% slow-release urea group; SBM, soybean meal; DM, dry matter; OM, organic matter; EE, ether extract; CP, crude protein; NDF, neutral detergent fiber; ADF, acid detergent fiber. ^2^ Contained per kg premix: vitamin A, 60,000 IU; vitamin D_3_, 6000 IU; vitamin E, 400 UI; Fe, 500 mg; Cu, 200 mg; Zn, 500 mg; Mn, 400 mg; I, 5 mg; Se, 2 mg.

**Table 2:** Primer sequence for real-time PCR.

Target Species	Forward/Reverse	Primer Sequence	References
Total bacteria	F R	CGGCAACGAGCGCAACCC CCATTGTAGCACGTGTGTAGCC	Denman and McSweeney [21]
Fungi	F R	GAGGAAGTAAAAGTCGTAACAAGGTTTC CAAATTCACAAAGGGTAGGATGATT	Denman and McSweeney [21]
*R. albus*	F R	CCCTAAAAGCAGTCTTAGTTCG CCTCCTTGCGGTTAGAACA	Wang et al. [22]
*R. flavefaciens*	F R	CGAACGGAGATAATTTGAGTTTACTTAGG CGGTCTCTGTATGTTATGAGGTATTACC	Denman and McSweeney [21]
*F. succinogenes*	F R	GTTCGGAATTACTGGGCGTAAA CGCCTGCCCCTGAACTATC	Denman and McSweeney [21]
*B. fibrisolvens*	F R	ACCGCATAAGCGCACGGA CGGGTCCATCTTGTACCGATAAAT	Stevenson and Weimer [23]
*Prevotella*	F R	GGTTCTGAGAGGAAGGTCCCC TCCTGCACGCTACTTGGCTG	Stevenson and Weimer [23]

**Table 3:** Effects of IBA and SRU as substitutes for SBM on 24 h in vitro rumen fermentation.

Items ^1^	Control	SRU	SRU-I	IBA-S	IBA	SEM	*p*-Value
Nutrient disappearance, %							
DM	58.9 ^c^	59.3 ^c^	61.2 ^b^	62.6 ^a^	62.5 ^a^	0.27	<0.01
CP	46.5 ^c^	50.9 ^a^	49.4 ^b^	46.6 ^c^	45.4 ^d^	0.23	<0.01
NDF	28.9 ^c^	29.3 ^c^	32.6 ^b^	37.0 ^a^	36.2 ^a^	1.07	<0.01
ADF	24.2 ^b^	25.0 ^b^	26.8 ^ab^	28.7 ^a^	28.9 ^a^	1.26	0.05
Total gas (mL/g DM)	131.4 ^c^	131.5 ^c^	133.9 ^b^	139.3 ^a^	139.1 ^a^	0.70	<0.01
Vmax (mL/g DM)	185.9 ^c^	189.2 ^c^	198.3 ^b^	211.5 ^a^	208.4 ^a^	3.59	0.02
S (/h)	0.050	0.049	0.047	0.050	0.051	0.004	0.74
L (h)	0.07	0.16	0.13	0.10	0.10	0.05	0.28
Fermentation parameters							
pH	6.68 ^a^	6.68 ^a^	6.67 ^ab^	6.66 ^b^	6.66 ^b^	0.01	<0.01
NH3-N (mM)	4.74 ^c^	5.08 ^a^	4.93 ^b^	4.72 ^c^	4.59 ^d^	0.05	<0.01
TVFA (mM)	27.8 ^c^	27.6 ^c^	28.9 ^b^	30.2 ^a^	30.1 ^a^	0.12	<0.01
VFA (mM)							
Acetate	15.2 ^c^	15.1 ^c^	16.2 ^b^	17.2 ^a^	17.1 ^a^	0.10	<0.01
Propionate	7.05	7.00	7.08	7.20	7.10	0.11	0.34
Butyrate	3.98	4.05	4.11	4.13	4.18	0.06	0.09
Isobutyrate	0.57 ^d^	0.55 ^d^	0.65 ^c^	0.74 ^b^	0.81 ^a^	0.03	0.01
Valerate	0.66	0.67	0.65	0.66	0.66	0.03	0.97
Isovalerate	0.31	0.20	0.27	0.29	0.23	0.06	0.67
A:P ratio	2.16 ^c^	2.17 ^c^	2.29 ^b^	2.39 ^a^	2.40 ^a^	0.04	0.01

^a, b, c, d^ Within a row, means without a common superscript differ at *p* ≤ 0.05. ^1^ SRU, 0.9% slow-release urea group; IBA, 0.9% isobutyramide group; SRU-I, 0.6% slow-release urea + 0.3% isobutyramide group; IBA-S, 0.6% isobutyramide + 0.3% slow-release urea group; SBM, soybean meal; DM, dry matter; CP, crude protein; NDF, neutral detergent fibre; ADF, acid detergent fibre; Vmax, the asymptote gas production; S, fractional degradation rate; L, lag phase; NH_3_-N, ammonia nitrogen; TVFA, total volatile fatty acids; VFA, volatile fatty acid; A:P ratio, ratio of acetate and propionate.

**Table 4:** Effects of IBA and SRU as substitutes for SBM on animal performance and nutrient apparent digestibility in beef cattle.

Items ^1^	Control	SRU	SRU-I	IBA-S	SEM	*p*-Value
DMI, kg/d	12.2	12.5	12.5	12.3	0.26	0.71
ADG, kg/d	0.96	0.95	1.00	1.07	0.04	0.09
Nutrient apparent digestibility, %						
DM	76.1 ^c^	75.8 ^c^	77.1 ^b^	79.1 ^a^	0.32	0.03
OM	77.5 ^c^	77.3 ^c^	79.3 ^b^	81.2 ^a^	0.30	0.01
CP	72.0	72.3	71.6	71.5	0.70	0.49
NDF	64.9 ^c^	64.9 ^c^	67.2 ^b^	69.1 ^a^	0.34	<0.01
ADF	53.7 ^c^	53.2 ^c^	56.6 ^b^	58.5 ^a^	0.44	<0.01

^a, b, c^ Within a row, means without a common superscript differ at *p* ≤ 0.05. ^1^ SRU, 0.9% slow-release urea group; SRU-I, 0.6% slow-release urea + 0.3% isobutyramide group; IBA-S, 0.6% isobutyramide + 0.3% slow-release urea group; SBM, soybean meal; ADG, average daily gain; DMI, dry matter intake; DM, dry matter; OM, organic matter; CP, crude protein; NDF, neutral detergent fibre; ADF, acid detergent fibre.

**Table 5:** Effects of IBA and SRU as substitutes for SBM on rumen fermentation in beef cattle.

Items ^1^	Control	SRU	SRU-I	IBA-S	SEM	*p*-Value
pH	6.49 ^a^	6.51 ^a^	6.37 ^b^	6.34 ^b^	0.02	0.02
NH_3_-N (mM)	12.9 ^c^	13.9 ^a^	13.5 ^b^	12.9 ^c^	0.11	<0.01
TVFA (mM)	96.1 ^c^	95.4 ^c^	99.6 ^b^	103.5 ^a^	1.29	0.02
VFA (mM)						
Acetate	60.9 ^c^	60.4 ^c^	63.0 ^b^	65.8 ^a^	0.73	0.04
Propionate	20.1	19.9	19.8	20.0	0.32	0.90
Butyrate	11.5 ^c^	11.5 ^c^	12.3 ^b^	13.2 ^a^	0.39	0.03
Isobutyrate	0.91 ^c^	0.87 ^c^	1.21 ^b^	1.45 ^a^	0.10	0.01
Valerate	1.25	1.28	1.18	1.31	0.09	0.52
Isovalerate	1.78	1.69	1.78	1.91	0.15	0.77
A:P ratio	3.01 ^c^	3.01 ^c^	3.21 ^b^	3.28 ^a^	0.02	0.01
MCP (mg/mL)	0.78 ^c^	0.78 ^c^	0.82 ^b^	0.84 ^a^	0.01	<0.01
Protozoa (log_10_/mL)	5.86	5.84	5.85	5.83	0.02	0.16
Enzyme						
Carboxymethyl cellulase (μmol glucose/h/mL)	3.53 ^c^	3.54 ^c^	3.67 ^b^	3.79 ^a^	0.04	<0.01
Xylanase (μmol xylose/min/mL)	1.42 ^c^	1.41 ^c^	1.47 ^b^	1.52 ^a^	0.01	<0.01

^a, b, c^ Within a row, means without a common superscript differ at *p* ≤ 0.05. ^1^ SRU, 0.9% slow-release urea group; SRU-I, 0.6% slow-release urea + 0.3% isobutyramide group; IBA-S, 0.6% isobutyramide + 0.3% slow-release urea group; SBM, soybean meal; NH_3_-N, ammonia nitrogen; TVFA, total volatile fatty acids; VFA, volatile fatty acid; A:P ratio, ratio of acetate and propionate; MCP, microbial crude protein.

**Table 6:** Effects of IBA and SRU as substitutes for SBM on the relative abundance of ruminal microbes in beef cattle (% of total bacterial 16S rDNA).

Items ^1^	Control	SRU	SRU-I	IBA-S	SEM	*p*-Value
Fungi	0.11	0.09	0.10	0.10	0.01	0.73
*R. albus* × 10^−2^	1.12	1.15	0.97	0.97	0.11	0.76
*R. flavefaciens* × 10^−3^	5.69	5.53	5.56	5.55	0.69	0.99
*F. succinogenes*	6.45	5.61	5.87	5.99	0.52	0.82
*B. fibrisolvens* × 10^−2^	0.93 ^c^	0.84 ^c^	1.21 ^b^	1.45 ^a^	0.06	<0.01
*Prevotella*	55.1	55.6	56.7	57.4	3.34	0.96

^a, b, c^ Within a row, means without a common superscript differ at *p* ≤ 0.05. ^1^ SRU, 0.9% slow-release urea group; SRU-I, 0.6% slow-release urea + 0.3% isobutyramide group; IBA-S, 0.6% isobutyramide + 0.3% slow-release urea group.

**Table 7:** Effects of IBA and SRU as substitutes for SBM on blood parameters in beef cattle.

Items ^1^	Control	SRU	SRU-I	IBA-S	SEM	*p*-Value
Nutrient index						
BUN (mmol/L)	4.21 ^b^	4.61 ^a^	4.49 ^a^	4.19 ^b^	0.11	0.03
TP (g/L)	62.7	62.7	62.5	63.2	2.54	1.00
GLU (mmol/L)	3.54 ^b^	3.50 ^b^	3.73 ^a^	3.69 ^a^	0.05	0.03
TG (mmol/L)	0.08	0.07	0.08	0.08	0.01	1.00
CHO (mmol/L)	2.09	2.14	2.13	2.12	0.27	0.99
Health index						
CREA (μg/L)	7463	7670	7463	7648	544.91	0.99
UA (μmol/L)	14.7	14.5	14.9	14.6	1.90	1.00
TMAO (µmol/L)	41.0	42.5	43.3	42.8	2.17	0.75
ALT (U/L)	19.5	20.7	20.6	20.4	0.87	0.79
AST (U/L)	62.1	60.6	62.6	62.9	2.10	0.87

^a, b^ Within a row, means without a common superscript differ at *p* ≤ 0.05. ^1^ SRU, 0.9% slow-release urea group; SRU-I, 0.6% slow-release urea + 0.3% isobutyramide group; IBA-S, 0.6% isobutyramide + 0.3% slow-release urea group; SBM, soybean meal; BUN, blood urea nitrogen; TP, total protein; GLU, glucose; TG, triglyceride; CHO, cholesterol; CREA, creatinine; UA, uric acid; TMAO, trimetlylamine oxide; ALT, alanine aminotransferase; AST, aspartate aminotransferase.

## Data Availability

Data are contained within the article.
